# Treatment decisions for older adults with advanced chronic kidney disease

**DOI:** 10.1186/s12882-017-0617-3

**Published:** 2017-06-19

**Authors:** Steven J. Rosansky, Jane Schell, Joseph Shega, Jennifer Scherer, Laurie Jacobs, Cecile Couchoud, Deidra Crews, Matthew McNabney

**Affiliations:** 1 Dorn Research Institute, WJBD VA Hospital, Columbia, SC USA; 2 Section of Palliative Care and Medical Ethics, Renal-Electrolyte Division, University of Pittsburgh School of Medicine, Pittsburgh, USA; 3 VITAS Healthcare, Miami, FL USA; 4 Division of Palliative Care and Division of Nephrology, NYU School of Medicine, New York, NY USA; 5 Department of Medicine, Albert Einstein College of Medicine, New York, NY USA; 6 REIN registry, Agence de la biomedicine, Saint Denis La Paine, France; 7 Division of Nephrology, Department of Medicine, Welch Center for Prevention Epidemiology and Clinical Research, Johns Hopkins Medical Institutions, Baltimore, USA; 8 Division of Geriatrics, Johns Hopkins University, Baltimore, MD USA

**Keywords:** Older adult, Dialysis, Shared decision, Conservative care, Comorbidity, Glomerulofiltration rate

## Abstract

Dialysis initiation rates among older adults, aged 75 years or greater, are increasing at a faster rate than for younger age groups. Older adults with advanced CKD (eGFR < 30 ml/min/1.73 m^2^) typically lose renal function slowly, often suffer from significant comorbidity and thus may die from associated comorbidities before they require dialysis.

A patient’s pattern of renal function loss over time in relation to their underlying comorbidities can serve as a guide to the probability of a future dialysis requirement. Most who start dialysis, initiate treatment “early”, at an estimated glomerulofiltration rate (eGFR) >10 ml/min/1.73 m^2^ and many initiate dialysis in hospital, often in association with an episode of acute renal failure. In the US older adults start dialysis at a mean e GFR of 12.6 ml/min/1.73 m^2^ and 20.6% die within six months of dialysis initiation. In both the acute in hospital and outpatient settings, many older adults appear to be initiating dialysis for non-specific, non-life threatening symptoms and clinical contexts. Observational data suggests that dialysis does not provide a survival benefit for older adults with poor mobility and high levels of comorbidity. To optimize the care of this population, early and repeat shared decision making conversations by health care providers, patients, and their families should consider the risks, burdens, and benefits of dialysis versus conservative management, as well as the patient specific symptoms and clinical situations that could justify dialysis initiation. The potential advantages and disadvantages of dialysis therapy should be considered in conjunction with each patient’s unique goals and priorities.

In conclusion, when considering the morbidity and quality of life impact associated with dialysis, many older adults may prefer to delay dialysis until there is a definitive indication or may opt for conservative management without dialysis. This approach can incorporate all CKD treatments other than dialysis, provide psychosocial and spiritual support and active symptom management and may also incorporate a palliative care approach with less medical monitoring of lab parameters and more focus on the use of drug therapies directed to relief of a patient’s symptoms.

## Background

Beginning in 1972, government funding for dialysis treatment and renal transplantation became available in the US. Over time the dialysis population shifted from a younger, healthier cohort to an older, more medically complex group of patients. Between 1980 and 2012 patients aged 65-74 initiating dialysis increased by 47% while those aged ≥ 75 (older adults) increased by 300% [1, 2]. Dialysis can be a life-extending treatment for patients of all ages, but one year mortality for older dialysis starts in the US was 41%, as compared to 28% for the those aged 65-74 and 17% for patients aged 45-64 [1]. In addition to limited life expectancy, many older adults experience functional decline and increased episodes of hospitalization after starting dialysis [2].

## Main text

Current nephrology guidelines recommend an age-neutral approach to chronic kidney disease (CKD) management based upon the level of estimated glomerular filtration rate (eGFR) and the presence of proteinuria [3]. Nephrology referrals are recommended for patients with estimated glomerulofiltration rate (eGFR) <30 ml/min/1.73 m^2^, abrupt sustained fall in eGFR (Acute Kidney Injury – AKI), albumin to creatinine ratio of >300 mg/gm. or rapid progression of renal failure, as defined by a sustained decline in renal function of >5 ml/min/1.73 m^2^ /year [3]. This approach may need to be modified, as there are large differences in prognosis and the trajectory of illness for older and younger adults with similar levels of eGFR [4]. As compared with younger adults, older adults with advanced kidney disease lose their renal function more slowly, have multiple other comorbidities, and face a substantially higher competing risk of death (from comorbidities) before being required to make a decision regarding the initiation of dialysis [5].

Dialysis initiation trends have led to earlier initiation, that is, starting dialysis at higher levels of eGFR. Because of this trend, many older adults who may have otherwise died from non-renal failure issues are faced with a decision regarding dialysis. Between 1996 and 2009 the percent of “early”, at eGFR >10 ml/min/1.73 m^2^, US dialysis starts in older adults increased from 25% to 62% [6]. Several recent observational studies using US and other countries’ dialysis registry data and one randomized controlled trial failed to demonstrate a survival benefit for “early start” dialysis [7]. These studies provide support for recent guidelines, which recommend deferring dialysis until patients have low levels of eGFR (≤6 ml/min/1.73 m^2^) unless a patient is symptomatic at a higher eGFR level [7, 8]. Although there is agreement that a patient’s symptoms should be the primary determinant for starting dialysis, eGFR remains a primary consideration for many nephrologists and symptoms that drive the decision to start dialysis are generally non specific and not life threatening. [7, 9–13]

While both eGFR and kidney failure related symptoms figure prominently in recent dialysis initiation guidelines, much less attention has been given to a patient’s goals and priorities. There is growing recognition that clinicians need to ensure maximal involvement of patients and their families in treatment decisions [14–19]. This shared decision-making is a process whereby patients and providers can discuss the benefits and burdens of potential treatment strategies in the context of each patient’s priorities and needs [20]. A major challenge for to this shared decision approach is the fact that many older adults initiate dialysis during acute illness, without the time to understand the potential advantages and disadvantages of starting dialysis versus a non-dialytic conservative management approach [15–18, 20–22]. Early and repeat discussions are necessary to address this challenge.

**Fig. 1:**
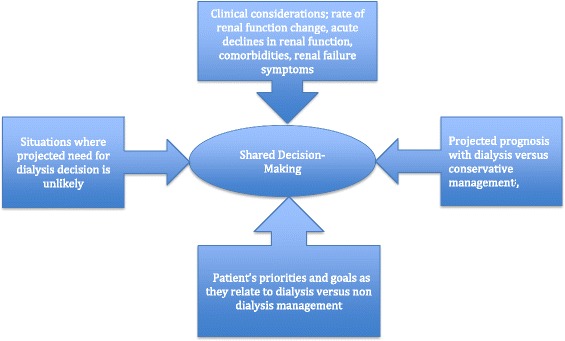
Framework for management of advanced CKD in older adults. The competing risk of death from non renal causes due to comorbidities and slow loss of renal function, < 3 ml/min/1.73 m^2^/year of eGFR [25, 28–30], makes the likelihood of the need for a dialysis decision low. Patient’s comorbidities and other parameters are used in tools for survival projections ([34, 35, 38–46] https://www.qxmd.com/calculate/calculator/3-month-mortality-in-incident-elderly-esrd-patients). High comorbidity and poor functional status may eliminate any dialysis survival advantage [2, 6, 14, 18, 44, 45]. A patient’s priorities and goals should be considered in conjunction with advantages and disadvantages of dialysis (listed in Table 2), in the shared decision process

The current review provides a pragmatic framework for the shared decision making process for older adults with advanced CKD [Fig. 1]. The questions addressed include: 1- how does a patient’s rate of loss of renal function, comorbidities and episodes of AKI, impact the likelihood that a dialysis decision will become necessary? 2- in what clinical situations should a non dialysis approach be considered? 3-How does AKI, as a precursor to chronic dialysis, relate to dialysis decisions? 4- what symptoms justify dialysis initiation in older adults? 5- how can clinicians help patients understand the potential benefits and harms of dialysis versus conservative management in the context of a patient’s symptoms, goals and priorities? Although the issues are complex, an open dialogue can help physicians understand what matters most to their patients. In turn the patient can gain greater control over decisions related to the management of their advanced CKD. When fully informed, some patients may opt for non-dialytic conservative management [Table 1] that can include all CKD therapies and may also include a palliative care emphasis, which prioritizes a patient’s comfort and symptom relief [15–22].

**Table 1:** Clinical considerations for discussions about dialysis versus conservative management^a^

Clinical Issues	Suggested Track^b^		Comments
	Dialysis^c^	Conservative^d^	
Renal Function Trajectory (RFD)			RFD defined as rate of decline of a patient’s estimated GFR (eGFR) per year^e^
Slow < 3 ml/min/1.73 m^2^ /year^f^			
Low Comorbidity^g^		□^h^	Patients are unlikely to be faced with a dialysis decision, but if their RFD increases, or they have an AKI episode, they may be good candidates for chronic dialysis.
High Comorbidity^i^		□□□	These patients are the most likely to remain in a conservative care track due to slow loss of renal function and high probability of death from comorbidity related issues.
Medium 3–5 ml/min/1.73 m^2^ /year^j^			
Low Comorbidity	❍❍		Compared with patients who have a slow RFD, these patients are more likely to require dialysis, especially if starting from an eGFR close to 15 ml/min/1.73 m^2^ (see Fig. 2).
High Comorbidity^i^		□□	Due to the relationship between faster RFD and worse survival [23, 36], these patients are likely to die before dialysis is required and therefore remain on a conservative track.
Fast >5 ml/min/1.73 m^2^ /year^k^			
Low Comorbidity	❍❍❍		These patients are the most likely to require dialysis and should be offered all treatment modalities, including renal transplant [2].
High Comorbidity		⧠⧠	Likelihood of remaining in conservative track may be low for most patients. Patient and family input with emphasis on a patient’s treatment goals is critical (Fig. 1, Table 2). Short survival on dialysis likely.
Acute Kidney Injury (AKI			Defined as patients who have a sudden sustained serum creatinine increase e [3] and most often uses a serum creatinine of ≥ 2x baseline creatinine [51]. Dialysis may in many cases be initiated “early” (eGFR > 10 ml/min/1.73 m^2^), [50, 52–54] and eGFR may overestimate true GFR [7, 52].
Low Comorbidity	❍❍		If patients have renal failure symptoms dialysis may be necessary. Preemptive dialysis, without a conventional dialysis indication, has not been shown to be beneficial [53, 54]. Recovery of renal function should be tracked [81, 82]
High Comorbidity		□□	Non-dialysis management should be considered during joint decision discussions due to a predicted short survival after dialysis initiation. Surrogate decision makers may choose dialysis if patients have not expressed a desire for non-dialysis management [19].

^a^This table is meant as a framework for ongoing joint decision conversations with older adults, defined as age ≥ 75, with advanced CKD, eGFR <30 ml/min/1.73 m^2^. Rate of loss of renal function, a patient’s comorbidity level, and episodes of acute declines in kidney function relate to the potential need for a dialysis decision and the choice of dialysis versus conservative management

^b^Suggested tracks are understood as choices that a patient may make with discussion and advice from the health care team. The tracks are meant to be flexible, since patients may have changes in rate of renal function loss, comorbidities, and may have single or multiple episode of acute renal failure as well as changes in their goals and priorities which may influence their desire to be managed with dialysis versus a conservative (non-dialytic) manner

^c^Unless otherwise stated, dialysis modality is hemodialysis. There is no definitive data on comparative elderly patient survival with hemodialysis versus peritoneal dialysis. Issues regarding dialysis modality choice and consideration for renal transplantation are discussed by Berger, et al. [2]

^d^The conservative track is conservative management, which includes shared decision making, active symptom management, psychosocial and spiritual support, treatment options that focus on a patients priorities which may include a palliative approach with a primary emphasis on relief of a patient’s symptoms, with less monitoring and pharmacologic therapy [15–21]

^e^RFD can be calculated using the arithmetic difference between first and last available eGFR or the first and last year’s average eGFR divided by the initial value [25–27]. Some limitations for this calculation include – non linear e GFR patterns, stability and increases of eGFR; episodes of acute renal failure are not considered [23, 24]

^f^Available studies suggest that the majority of elderly advanced CKD patients have a slow loss of eGFR, < 3 ml/min/1.73 m^2^/year [25, 28–30]

^g^Most clinicians would consider a **minimum** projected survival > 1 year for older adults with advanced CKD as low comorbidity. Several prognostic scores have been developed to predict which patients will require dialysis [34, 35, 38] and to predict post dialysis initiation survival [39–46], including an on line calculator (https://www.qxmd.com/calculate/calculator/3-month-mortality-in-incident-elderly-esrd-patients). The parameters used to predict short survival after dialysis initiation include: poor functional status (i.e., inability to transfer), nursing home residence, low serum albumin (<2.5 gm/dl), low body mass index (<18.5 kg/m^2^) significant heart failure (New York Heart Association grade 3, 4), severe peripheral vascular disease, dementia, and a negative response to the “surprise question” (would I be surprised if this patient died in the next twelve months?)

^h^One, two, three squares or circles are used to approximate the weight of the suggested approach for a patient to consider --a conservative or dialysis care track

^i^Most clinicians would consider a projected survival of <3 months to represent high comorbidity but for some, a 6 month projected survival would qualify. An on-line calculator is available to identify patients with projected 3-month mortality (https://www.qxmd.com/calculate/calculator/3-month-mortality-in-incident-elderly-esrd-patients). Other prognostic scores can be used to help predict a high 3 and 6-month dialysis mortality [40, 43, 44, 46]. Additionally, the following situations may be considered for high comorbidity classification:

A. Dialysis cannot be provided safely [19, 47]

a. Patient needs to be restrained or heavily sedated to use his vascular access

b. Patient unable to cooperate due to dementia

c. Multiorgan failure with profound hypotension

B. Incurable malignancy or other non-renal cause of imminent death [19].

C. Older adults with ≥ 2 of the following conditions [47]

a. High comorbidity score

b. Significantly impaired functional status

c. Severe chronic malnutrition (serum albumin <2.5 g/dL)

d. Clinician’s response of “no” to surprise question -“I would not be surprised if the patients dies within the next year”

D. Patient is dependent on artificial hydration and nutrition to survive

^j^Medium rate of renal function loss is included for completeness and is not used in published accounts of RFD

^k^A fast RFD has generally been reported for most patients who start dialysis [23, 25, 30, 48]

### Clinical considerations for dialysis versus conservative management decisions [Table 1]

#### Rate of loss of renal function and the potential need for dialysis

Some older adults, when informed that they have advanced CKD, may assume that dialysis is inevitable [Fig. 1]. This diagnosis may be the result of a single eGFR that may not be reflective of the severity (a repeat eGFR may be lower) or the course of a patient’s kidney disease. Patterns of eGFR may reflect intervals of stability, increases, decreases and slow or fast rates of change [23, 24]. The rate of decline in a patient’s eGFR (their renal function decline, RFD) may be more important in determining a patient’s prognosis than any single eGFR measure [3, 25]. Patterns of eGFR change are usually determined by slope analysis (least squares and Bayesian methodologies) [23–25]. As slope based methodologies to determine rates of renal decline are not readily available to clinicians, a simpler calculation uses a patient’s initial and final or the average of first and last year’s eGFRs to calculate their change in eGFR per year [Table 1] [25–27]. This estimate makes several assumptions: a) that eGFR declines (increases and stable e GFRs are not uncommon; b) that eGFR approximates true GFR (this assumes stable muscle mass and the lack of an unusual dietary pattern or body habitus), c) that the eGFR declines linearly (non linear patterns may occur in 40% of patients [24]); and c) that patients do not have episodes of AKI, during the measurement interval. Using this simple calculation, clinicians can determine whether a patient has a slow (<3 ml/min/1.73 m^2^/year) medium (>3 and <5 ml/min/1.73 m^2^/year) or fast (≥5 ml/min/1.73 m^2^/year) RFD (normal older adult RFD is approximately 1 ml/min/1.73 m^2^/year) as one factor that may relate to a future need for dialysis [Table 1].

Most older adults with advanced CKD lose renal function at a slow rate, and two thirds may have stable renal function for several years [25, 28–30]. This slow rate of loss may relate to the fact that proteinuria is the main determinant of a fast RFD [31] and that low proteinuric vascular nephropathy may account for 39% of the causes of advanced CKD in older adults [32]. Nonproteinuric CKD with stable eGFR may be a common pattern for many older adults [33].

### Use of a patient’s rate of renal function decline and intensity of comorbidity, to help predict need for dialysis and post dialysis initiation survival

In addition to estimating the rate of a patient’s renal function change, assessment of a patient’s level of comorbidity is another important determinant of whether a patient will face a dialysis decision. Few validated risk prediction models are available to identify which older adult advanced CKD patients will require dialysis [34, 35]. A fast RFD is connected to both a greater likelihood of reaching a low eGFR where dialysis may be considered as well as a worse survival [25, 36]. None of the existing prediction models incorporate RFD; comorbidity related competing risk of death prior to a dialysis requirement, or AKI episodes.

**Fig. 2:**
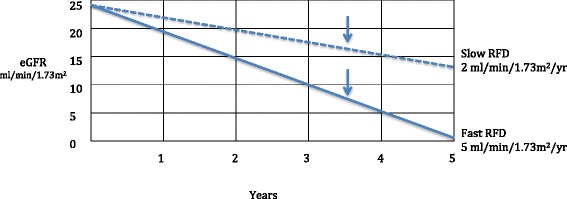
Use of estimated rate of renal function decline (RFD) and survival to help plan for future dialysis needs. Suggested method of calculation of RFD, see Table 1. Hypothetical 75 year olds with baseline eGFR of 25 ml/min/1.73 m^2^, one with slow RFD, *dotted line*, and one with fast RFT, *solid line*. In contrast to fast RFD patient, slow RFD patient unlikely to face dialysis decision [25, 28–30]. *Vertical arrow* indicates a projected survival of 3.5 years [37]

A patient’s historical rate of renal function loss, combined with an estimate of their survival, may be useful to help determine the likelihood that they will face a dialysis decision [Fig. 2, Table 1]. Using data from a Canadian cohort of patients with advanced CKD, average projected survival (without comorbidity adjustments) for patients ages 75, 80 and 85 years with a starting eGFR of 15-30 ml/min/1.73 m^2^ is approximately 3.5, 2.8 and 1.5 years, respectively [37]. Using this Canadian data we can assume that a 75-year-old patient has a projected 3.5-year survival. In addition [Fig. 2] this hypothetical patient has a linear RFD, no AKI episodes and a starting eGFR of 25 ml/min/1.73 m^2^. If this patient has a fast RFD (≥5 ml/min/1.73 m^2^) he will reach an eGFR where dialysis is likely to be considered. On the other hand, if the patient has a slow RFD (2 ml/min/1.73 m^2^), he is unlikely to require a dialysis decision [Fig. 2]. This approach and the application of different baseline eGFRs, RFDs and survival projections, can offer patient information on whether they are likely to face a dialysis decision.

Once a patient is faced with this decision, several validated comorbidity based tools have been designed to predict post dialysis initiation survival [Table 1], ([38–46] https://www.qxmd.com/calculate/calculator/3-month-mortality-in-incident-elderly-esrd-patients). Those patients with low comorbidity levels and a predicted survival of more than three years, should be considered for all renal failure treatment modalities, including renal transplantation [2]. In contrast to these healthy older adults, patients with a high three and six month expected mortality may choose to delay initiation and may be candidates for non dialysis conservative management. A study of US older adult incident dialysis patients, 2009-2010, demonstrated a mean starting eGFR of 12.6 ml/min/1.73 m^2^, and a three and six month’s mortality of 12.4% and 20.4%, respectively [44]. Data from this study and a recent report from France show that one third of older adults initiating dialysis with poor functional status, as defined by strong dependency in activities of daily living, inability to ambulate or presence of an amputation, died within three months of dialysis initiation [44, 45]. Other factors associated with poor short-term survival include a high comorbidity index score [41–46], nursing home residence [44], low serum albumin [40, 41, 44, 45], low body mass index [42], significant heart failure [39, 44], and a negative response to the “surprise” question asked of the nephrologist (would I be surprised if this patient died in the next twelve months?) [43, 57]. Identification of patients with high three-month mortality (and thus candidates for conservative therapy, [Table 1]), can be assisted using on-line tools (https://www.qxmd.com/calculate/calculator/3-month-mortality-in-incident-elderly-esrd-patients). In addition to the high risk conferred by poor functional status and high comorbidity, older adults who initiate dialysis in the acute care setting may also have high short-term mortality rates [47–50].

### AKI and the dialysis decision [Table 1]

Shared decision making for older adults deciding about electively initiating dialysis is difficult. However, it is even more challenging to decide whether to initiate dialysis for older adults who have an episode of AKI during a hospitalization [Table 1] [51–54]. Although the majority of older adults with advanced CKD lose renal function slowly, 51% of an older adult (mean age 77) US dialysis population had an episode of AKI in the six months prior to starting dialysis [55] and 65% of patients in this age group started dialysis while hospitalized [49].

Acute declines in kidney function prior to dialysis initiation are not reported to the US dialysis registry. Completion of registry data is linked to Medicare coverage, which begins after the first ninety-day days of dialysis treatments. Thus, survival data for patients who die in this interval may not be captured and thus three-month mortality rates may be underestimated, [Table 1] [56]. Nevertheless, compared with elective starts, patients who initiate dialysis during emergent situations are likely to have a higher initial eGFR, a higher level of comorbidity (including episodes of congestive heart failure) and thus may experience higher ninety-day mortality rates [45, 49–54].

Since acute dialysis in hospital is a common scenario for older adults, early advanced care planning discussions should include conversations about emergent dialysis as one of the life support options. If given the choice before an emergent situation occurs, some of these patients may choose conservative management, [Table 1], [17, 20, 21, 57, 58]. Others may opt for a time limited trial of dialysis [59]. In both the acute and chronic dialysis initiation settings, a dialysis trial has been suggested as a way to give the patient an opportunity to assess the wisdom of pursuing chronic dialysis [20, 59]. For all patients who initiate dialysis after AKI and/or who opt for a trial of dialysis, monitoring of a patient’s residual renal function (by measures of interdialytic creatinine and or urea clearance) should be part of their care [7].Patients may lose 10% per month on dialysis, of their remaining endogenous renal function [7]. With this loss of endogenous renal function, discontinuation of dialysis could potentially result in death sooner than if a dialysis trial were not chosen. On the other hand, monitoring of post dialysis initiation renal function (especially after AKI starts) may show that a patient’s renal function has improved to the point where they can discontinue dialysis [7, 51, 52, 81, 82].

In an acute care setting, delaying dialysis may not be an option for patients with a rapid decline in renal function and associated oliguria. Use of serum creatinine based measures of renal function in these situations may be confounded by the decline in somatic protein stores in acutely ill patients [52]. On the other hand, “early” (absent a conventional or life threatening indication) dialysis initiation in the acute setting is not supported by available studies [52–54]. As these acute episodes are common but not predictable, repeated joint decision discussions are necessary to have an understanding of a patient’s preferences before these stressful, often intensive care unit related decisions, are required [58, 59].

### The dialysis decision as it relates to patient symptoms

Recent guidelines for dialysis initiation have suggested a greater emphasis on a patient’s symptoms, rather than a specific eGFR level, as the primary factor to consider when deciding whether to initiate dialysis [7]. In many cases, symptoms that precipitate dialysis initiation may be more of a consequence of older adult comorbidities [11] than their level of renal function [7, 45, 60]. The conventional indications to initiate acute or chronic dialysis include symptomatic refractory volume overload, especially if associated with oligoanuria; uremic pericarditis; refractory hyperkalemia; and severe acidosis [3, 7, 53, 54]. Many international guidelines consider nutritional deterioration, which is refractory to dietary intervention, a reason to initiate dialysis [7, 60]. This indication could be questioned since several large studies demonstrate progressive nutritional deterioration for both new onset and existing dialysis populations in association with a dialysis related increase in inflammation [7, 61].

Studies have indicated that fatigue and non-specific GI symptoms, including nausea and decreased appetite and not the “conventional indications”, were the reasons for the majority of decisions to start dialysis [9–12]. One study reported that the decision to start dialysis was made weeks or months before dialysis was actually initiated and often appeared to be solely based on eGFR [10]. Inpatient starts were often for cardiopulmonary symptoms (volume overload) while hyperkalemia accounted for only 3% of in-patient dialysis starts [10, 12]. In a prospective study of nursing home residents, 18% started dialysis at an eGFR > 15 ml/min/1.73 m^2^ and the majority of the new starts did not have any of the following dialysis indications (according to study design): volume overload, cognitive decline, weight loss, or a decline in the performance of activities of daily living (ADL) [11]. The latter indication may not be reasonable as older adults experience functional deterioration after the initiation of dialysis [2]. Even with the potential for eGFR to overestimate true GFR for older adults, non-specific symptoms of nausea, anorexia, and functional deterioration in measures of ADL probably do not justify dialysis initiation [7]. If given the option during shared decision-making discussions, many older adults may opt to delay dialysis until they have a conventional indication [2, 7].

### Shared decision-making regarding dialysis versus conservative management

Shared decision- making discussions are best initiated when patients are healthy enough to participate and share their goals and priorities, especially if their health condition should worsen. Outlining the situations that a patient would not want to undergo life-prolonging therapies such as dialysis can guide surrogate decision–makers who often face these difficult decisions when patients are too ill or cognitively impaired to do so themselves. Helping surrogate decision–makers follow their loved one’s wishes may help to decrease family member and health care team conflicts, especially during acute hospitalizations and sudden declines in a patient’s clinical and renal status [20].

Current evidence suggests that the decision to start dialysis is often driven by physician preference rather than a shared conversation in which the informed patient is the decision maker [2, 14–18, 62]. A discussion of a patient’s pattern of renal function loss over time in relation to their underlying comorbidities can serve as a guide to the probability of a future dialysis requirement [Figs. 1 and 2, Table 1].

Many patients regret deciding to start dialysis. It is possible that better advanced care planning may decrease this situation [15–18, 63–65]. In addition to a consideration of dialysis, patients must be given the option of conservative management [47]. This approach can incorporate all CKD treatments other than dialysis, provide psychosocial and spiritual support and active symptom management and may also incorporate a palliative care approach with less medical monitoring of lab parameters and more focus on the use of drug therapies directed to relief of a patient’s symptoms [1, 15, 63–66]. These non dialysis options are not only appropriate considerations for patients with high levels of comorbidity and poor functional status but have also been advocated for patients who are dependent on artificial hydration and nutrition to survive, are in a persistent vegetative state, have an incurable malignancy, or other non-renal causes of imminent death and for patients who can not be dialyzed safely [Table 1] [19]. Non-dialysis options may also be appropriate for patients whose goals and priorities are to focus on the quality of their life rather than treatments aimed to extend life. Although there are no prospective studies comparing survival with dialysis versus conservative management most studies and a recent meta-analysis showed similar survival [67–70]. Patients who choose conservative therapy have relatively preserved functional status until the last months of life [67–69].

Eliciting each patient’s goals and priorities is essential for shared decision making. These patient specific issues should be individually addressed when discussing the potential risks and benefits of a dialysis versus a non-dialysis approach [Table 2]. Some patients may prioritize maximizing life expectancy. Dialysis may provide life saving treatment for acutely ill oligoanuric patients, but is unlikely to provide a survival advantage for patients with a high comorbidity burden [Table 1] [2, 18, 53, 67, 68, 71]. Some older adults with high comorbidity, with a projected survival on dialysis of three to six months [44, 45], when informed of the potential disadvantages of dialysis, may opt for conservative care [16]. These disadvantages include irreversible loss of a patient’s residual renal function (which has a survival benefit), with a potential need for stricter fluid restriction [7, 60]. Other dialysis risks include cardiac ischemia, progressive left ventricular systolic dysfunction, increased frequency of sudden death and stroke [7, 72] and a high rate of withdrawal from dialysis, especially if dialysis is initiated early [73]. Finally, the likelihood of dying in a hospital (versus at home or in a hospice setting) is much higher for patients who choose dialysis compared to conservative management [2, 18].

**Table 2:** Potential advantages and disadvantages of choosing dialysis versus conservative management

Potential advantages of dialysis	Potential disadvantages of dialysis
• Possibly longer survival [67, 69–71] • May improve appetite • May be life saving in some AKI situations • Social contact/interactions with dialysis staff and patients	• Multiple painful access procedures [74–76] • Loss of residual renal function [7, 60] • Dialysis related fatigue hypotension, cardiac ischemia, and functional decline [7, 18, 60, 77] • Increased risk of sudden death and stroke [7, 72] • Time lost to dialysis and hospitalizations [18] • High mortality rate, first 3 months [7, 44, 45] • More likely to die in hospital versus conservative management [2, 18] • High discontinuation rates [73]

For some patients, the goals of starting dialysis may be to obtain relief of renal failure related symptoms and other quality of life issues [Table 2], [63–65]. Dialysis may facilitate treatment of intractable volume overload, improve physical symptoms such as a shortness of breath and decreased appetite, and can facilitate management of elevated potassium [Table 2]. The social interactions with staff and other patients during dialysis may significantly improve a patient’s quality of life. On the other hand, these purported benefits of dialysis may be less important than the patient’s desire to avoid the pain and discomfort associated with dialysis therapy. Older adults often require repeated painful vascular access procedures and some may die before the access is used [2, 74–76]. Central venous catheter dialysis access has been suggested as a way to mitigate some of the vascular access related discomfort, especially for patients with high comorbidity and a short predicted survival [74]. Home peritoneal dialysis is another option for some patients whose primary goal is freedom from pain [2]. Other than pain, potential adverse quality of life issues related to the dialysis choice include accelerated functional and cognitive declines as well as post dialysis fatigue and the feeling of being “washed out” after a dialysis treatment [2, 77]. Another relevant consideration is the time commitment required for dialysis. Some patients may be willing to sacrifice several months of longevity to gain more personal freedom [18], especially when considering that a large segment of their remaining life will be spent on dialysis and with dialysis related complications [2].

## Conclusions

In conclusion, older adults with advanced CKD are likely to die from non-renal failure related conditions before they are faced with a decision concerning dialysis [47]. Exceptions to this scenario are patients who lose renal function rapidly and have limited comorbidities. In the US, older adults initiate dialysis early (at eGFR > 10 ml/min/1.73 m^2^) and have a high comorbidity burden. Other countries, including Canada and New Zealand, have much lower rates of renal replacement therapy for older adults compared to the US [78]. Although dialysis initiation guidelines emphasize the presence of renal failure symptoms as justification to start dialysis, many older adults start dialysis preemptively, in the face of non-specific, often comorbidity related symptoms. These new dialysis patients are generally not informed about non-dialysis, conservative management options. To remedy this situation, an interdisciplinary team effort by health care providers [79], should consist of early and repeated discussions with patients and their families regarding a patient’s preferences and goals in the context of the potential benefits and harms of dialysis initiation. Conservative management may be a reasonable choice for patients whose primary goal is to maintain their independence and to avoid the time, pain, and discomfort related to dialysis, as well as for patients with poor functional status and a predicted post dialysis initiation projected survival of less than three months. Future studies should examine knowledge, attitudes and decision support interventions that could benefit older adults and their providers when making decisions regarding management of advanced CKD [66, 79, 80]. As well, more data is needed on outcomes of conservative and dialytic management and characteristics of older adults who would benefit from each of these approaches.

## Abbreviations

ADL: Activities of daily living
AKI: Acute kidney injury/acute renal failure
CKD: Chronic kidney disease
eGFR: Estimated glomerulofiltration rate
RFD: Rate of renal function decline
